# Safranal Prevents Liver Cancer Through Inhibiting Oxidative Stress and Alleviating Inflammation

**DOI:** 10.3389/fphar.2021.777500

**Published:** 2022-02-01

**Authors:** Youssef Abdalla, Ali Abdalla, Alaaeldin Ahmed Hamza, Amr Amin

**Affiliations:** ^1^ Department of Kinesiology, Michigan State University, East Lansing, MI, United States; ^2^ Weinberg Institute for Cognitive Science, University of Michigan, Ann Arbor, MI, United States; ^3^ Hormone Evaluation Department, National Organization for Drug Control and Research, Giza, Egypt; ^4^ The College, The University of Chicago, Chicago, IL, United States; ^5^ Biology Department, UAE University, Al Ain, United Arab Emirates

**Keywords:** liver cancer, prevention, safranal, oxidative stress, inflammation

## Abstract

Despite all efforts, an effective and safe treatment for liver cancer remains elusive. Natural products and their derived biomolecules are potential resources to mine for novel anti-cancer drugs. Chemopreventive effects of safranal, a major bioactive ingredient of the golden spice “saffron”, were evaluated in this study against diethylnitrosamine (DEN)–induced liver cancer in rats. Safranal’s mechanisms of action were also investigated in the human liver cancer line “HepG2”. When administered to DEN-treated rats, safranal significantly inhibited proliferation (Ki-67) and also induced apoptosis (TUNEL and M30 CytoDeath). It also exhibited anti-inflammatory properties where inflammatory markers such as NF-kB, COX2, iNOS, TNF-alpha, and its receptor were significantly inhibited. Safranal’s *in vivo* effects were further supported in HepG2 cells where apoptosis was induced and inflammation was downregulated. In summary, safranal is reported here as a potent chemopreventive agent against hepatocellular carcinoma that may soon be an important ingredient of a broad-spectrum cancer therapy.

## Background

Cancer is one of the most common causes of illness and death worldwide. In 2015, there were 17.5 million recorded cases of cancer and 9.5 million deaths from the disease, making it the second highest cause of death globally after heart disease (Zhou et al., 2016; Bray et al., 2018). Liver cancer is considered the fifth cause of death from cancer worldwide and the death rate from liver cancer represents 70% of deaths in African and Asian countries (Chessum et al., 2015; Plummer et al., 2016; Bray et al., 2018). In Egypt, for example, hepatocellular carcinoma (HCC) is responsible for about 14.8% of all cancer fatalities (Aglan et al., 2017). There are a number of factors that lead to the spread of HCC in Asia and Africa, for instance, chronic hepatitis (B and C) infection, chronic alcohol consumption, exposure to environmental pollutants such as aflatoxin, and environmental carcinogens including nitrosamines (Plummer et al., 2016; Zhou et al., 2016). Diethylnitrosamine (DEN) is considered an environmental carcinogen that we are exposed to daily since it is a component in processed food, cosmetics, gasoline, and tobacco (Park et al., 2009; Hayden and Ghosh, 2014; Tolba et al., 2015; Santos et al., 2017). Moreover, thanks to the human resemblance of lesions it induces in rats, DEN is often used to study different types of benign and malignant tumors in humans (Li et al., 2005). In fact, the initiation-promoting cancer development model, used in this study, mimics the early events of the latent period of human carcinogenesis (Tolba et al., 2015; Santos et al., 2017). The initiation of HCC can be produced by the administration of a single dose of DEN, a carcinogen that causes DNA ethylation and mutagenesis, and the biotransformation of normal hepatocytes into initiated cells (Santos et al., 2017). To expedite HCC development, exposure to a tumor promotor such as such as 2-acetyl aminofluorene (2-AAF) helps developing altered hepatocytes foci (AHF) and hyperplastic nodules and the final progression into HCC (Tolba et al., 2015; Santos et al., 2017).

Considering their great efficacy and low toxicity, natural substances and plants have been extensively studied and proposed as a chemoprotective therapy for many diseases (Amin and Mahmoud-Ghoneim, 2011; Koh et al., 2020; Hamza et al., 2021). Anti-cancer medicinal plants are used for several reasons; they contain nutritional and anti-tumor compounds, are able to delay or prevent cancer onset, can boost physiological status and the immune system (Block et al., 2015; Zhou et al., 2016; Koh et al., 2020), and most importantly, they represent a great alternative to conventional cancer treatments by decreasing or even eliminating side effects (Zhou et al., 2016). Consequently, anti-oxidative, anti-inflammatory, and hepatoprotective properties possessed by some natural compounds qualify them as potential candidates to protect against tumor initiation and growth (Zhou et al., 2016). The use of natural-based materials (Baig et al., 2019, Benassi et al., 2021) and phytochemicals that are low in toxicity and effective at the beginning of exposure to carcinogens is considered the most successful strategy available to protect against the development of liver cancer (DiMarco-Crook and Xiao, 2015; Maru et al., 2016).

Saffron is one of the oldest spices that was used since ancient times in Egypt and Rome as both a remedy and a culinary spice (Rios et al., 1996; Winterhalter and Straubinger, 2000). It consists of the dried stigmas of *Crocus sativus* flowers and is also one of the most commonly used species in folklore medicine to treat depression, asthma, and smallpox (Siddique et al., 2020). Saffron and its active substances have been reported to have anti-cancer, anti-oxygenic, and anti-inflammatory effects (Abdullaev, 2002; Abdullaev and Espinosa-Aguirre, 2004; Das et al., 2010; Amin et al., 2011; Amin et al., 2016; Ashktorab et al., 2019). For example, the presence of saffron and all its major constituents, such as crocin, crocetin, and safranal, were found to have significant anticancer activity in various tumors including prostate cancer, cervical cancer, leukemia, lung cancer, and liver cancer (Amin et al., 2011; Amin et al., 2016; Milajerdi et al., 2016; Mollaei et al., 2017; Khorasanchi et al., 2018). Similarly, saffron and the active substance crocin reduced the incidence of HCC induced by DEN and promoted by 2-acetyl aminofluorene (2-AAF) in rats (Amin et al., 2011; Amin et al., 2016; Amin and Awad, 2021) as well as reducing the incidence of stomach cancer induced by methyl-3-nitro-1-nitrosoguanidine in rats (Bathaie et al., 2013). Safranal, which gives saffron its aroma (Bathaie and Mousavi, 2010; Khorasanchi et al., 2018), was found to have dose-dependent antitumor effects against Hela and MCF7 cells (Malaekeh-Nikouei et al., 2013), prostate cancer cells (Samarghandian and Shabestari, 2013), and liver HepG2 cells (Al-hrout et al., 2018). In addition to its anti-cancer effects, this monoterpene also has antioxidant and anti-inflammatory properties in different animal models (Nanda and Madan, 2021). Despite all of its anti-tumor activities *in vitro*, the effects of safranal alone on an HCC model is yet to be determined and the mechanism that mediates its anti-cancer effect has yet to be fully understood.

The present experiments were designed to study the potential chemopreventive effects of safranal in a well-described model of HCC in rats, which was induced by DEN and promoted by 2-acetyl aminofluorene (2-AAF) during the early stages of hepatocellular tumor promotion. When promoting carcinogenesis experimentally, the AHF serve as pre-neoplastic indicators of HCC, weeks or months prior to its emergence. This strongly resembles the progression of human hepatocarcinogenesis (Li et al., 2005). In this animal model, the mechanisms of safranal’s different effects, such as its antioxidant, pro-apoptotic, anti-proliferative, and anti-inflammatory effects, were investigated and key regulators of different pathways were assessed. HepG2 cells were also used to assess safranal’s affects in human liver cancer cells.

## Materials and Methods

### Reagents and Materials

DEN, 2-AAF, 5,5′-dithiobis-(2-nitrobenzoic acid), thiobarbituric acid, Folin’s reagent, pyrogallol, SOD enzyme, H_2_O_2_, and bovine albumin were obtained from Sigma Chemical Co. (St. Louis, MO). Primary antibodies of Ki-67, COX-2 (Clone SP 21), iNOS (Ab-1), and NF-kB-P65 (Rel A, Ab-1) were purchased from Thermo Fisher Scientific, Anatomical Pathology, Fremont, USA (1:100 dilutions). GST-p form was obtained from Medical and Biological laboratories Co., Tokyo, Japan (1:1,000 dilution). M30 CytoDeath was purchased from Enzo life Science, USA. Anti-CD68 (ED1), -CD163 (ED-2) (1:300 dilution), and -phosphorylated form of tumor necrosis factor alpha receptor 1 (p-TNFR) (1:200 dilution) antibodies were obtained from Santa Cruz, CA, USA. Safranal (W338907 Aldrich) was obtained from Sigma-Aldrich, USA. CellTiter-Glo luminescent Cell viability assay kit and Caspase-Glo 3/7 luminescent assay kit were obtained from Promega (Woods Hollow. Rd., Madison Wisconsin, USA). HDAC Colorimetric Assay Kit and Human IL-8 ELISA Kit (EZHIL8) were obtained from the Millipore Corporation (28820 Single Oak Drive, Temecula, CA 92590, USA) and the TNF-α ELISA kit came from R&D Systems (Minnesota, USA).

### Animals

The experiment was conducted on 30 healthy albino Wistar rats, weighing 180–200 g, and aged 10–12 weeks. They were obtained from the animal house of the College of Medicine, Emirates University, after the protocol was approved by the Animal Research Ethics Committee, UAE University. All efforts have been made to reduce the suffering of animals and the number of animals used. The animals were placed in an air-conditioned room and were randomly distributed across five polycarbonate cages lined with wood chip bedding that was changed daily. The animals were placed under controlled conditions with a temperature of 22–24°C and a 12-h light/dark cycle throughout the experiment. Rats were provided with free access to standard pellet diet and tap water *ad libitum* and were acclimated to the environmental conditions for 2 weeks before the experimental procedure.

### Hepatocarcinogenesis Model

The development of the experimental hepatocarcinogenesis was carried out according to the protocol used by Espandiari et al. (2005) and Santos et al. (2015). In this protocol, the formation of cancer cells is initiated by an intraperitoneal injection of a single dose of the carcinogenic DEN (200 mg/kg b wt.) dissolved in saline. The neoplastic cell division was promoted with 5 days fasting-refeeding followed by the use of the promoting agent, 2-acetylaminofluorene (2-AAF). Fasting-refeeding and employing 2-AAF after 2 weeks of using DEN are reported as mitotic proliferative stimuli (Hayden and Ghosh, 2014). 2-AAF was introduced in the form of intra-gastric doses given daily for 6 days and then weekly for 4 weeks (30 mg/kg in 1% Tween 80).

### Experimental Design

**FIGURE 1:**
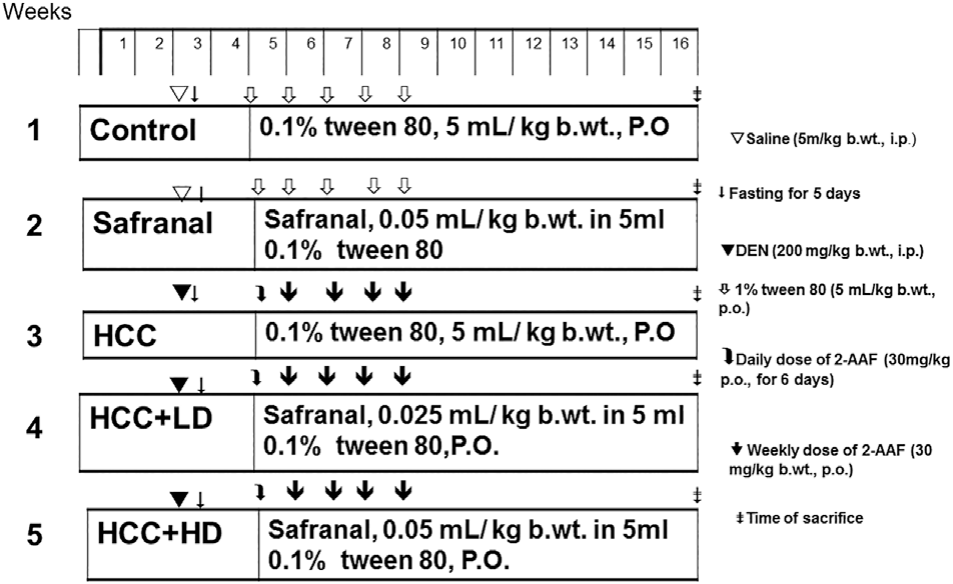
Schematic diagram showing the experimental design to induce and treat liver cancer *in vivo* study.

Safranal suspended in 0.1% (w/v) Tween 80 was administered orally at doses of 0.025 ml and 0.05 ml/kg b wt. to rats. The two doses used in this experiment are non-toxic and were shown to have anti-inflammatory and anti-oxidant effects in previous chemically induced inflammation and oxidative stress in rats (Hosseinzadeh and Sadeghnia, 2007; Hariri et al., 2011; Hosseinzadeh et al., 2013; Xue et al., 2020). A total of 30 adult male albino Wistar rats were randomly divided into five groups (n = 6) and subjected to different treatments. Group 1 (control) was orally administered the daily dose of distilled water containing 0.1% Tween 80 (5 ml/kg b wt.) (Safranal vehicle) throughout the experimental duration and were also injected with a single dose of saline (DEN vehicle). Group 2 (Safranal only) was subjected to a daily dose of safranal (0.05 ml/kg) through oral administration for the duration of the experimental period. Hepatocarcinogenesis was induced by DEN and promoted by 2-AAF, as reported previously, in group 3 (HCC). Rats in protective groups (groups 4–5) were treated with daily low/high doses of safranal at the beginning of promotion periods and continued for 12 weeks. The low dosage treatment with safranal consisted of 0.025 ml/kg and the high dosage treatment consisted of 0.05 ml/kg. The experimental design is illustrated in Figure 1.

### Sample Preparation

After 14 weeks of treatment with DEN and safranal, all rats were anesthetized and blood was collected via retro-orbital puncture under the influence of diethyl ether 24 h after last treatment. Then the rats are sacrificed under the influence of the diethyl ether by a cervical dislocation. Rat livers were rapidly dehydrated, washed with ice-cold normal saline solution, and then dried with blotting paper. Liver slices from different lobes were immediately fixed in 10% buffered formalin for histological and immunohistochemical analyses, and the other parts were rapidly frozen in liquid nitrogen and kept at −80°C for bio experiments. The serum was separated from the blood samples by centrifugation at 3,000 rpm for 20 min (4°C). Frozen liver samples are ground in ice-cold 150 mM Tris–HCl buffer (pH 7.4) with a 1:10 wt/v ratio (Hamza et al., 2018). Aliquots were prepared for the purpose of biochemical marker determination. Fixed tissue samples were processed and embedded in paraffin before being sectioned off into 5-μm sections. The fixed sections were placed onto glass slides and a routine staining by H&E was performed prior to examination under light microscope (Olympus DP71) (Hamza et al., 2018).

### FAH Formation and GST-p Expression

Histological examinations of the livers such as the classical foci of altered hepatocytes (FAH) were performed using an Olympus DP71-light microscope. FAH are characterized by the presence of a group of pale hepatocytes that are irregular in shape and contain large vacuoles in the cytoplasm with large hyperchromatic nuclei (Jiang et al., 2018). The presence of these precancerous changes was confirmed by immune histochemical detection of GST-p, which is a true indicator for quantitation of FAH (Satoh, 2018). The number and areas (mm^2^) of foci /cm^2^ of liver sections were estimated using a magnification of ×100. GST-p foci larger than 15 cells were considered and measured by using color image analysis software (NIS Elements Basic Research, version 3; Nikon, USA).

### Antioxidant Status in Liver

For determination of catalase activity CAT, a method by Aebi was followed (Aebi, 1984). CAT decomposes hydrogen peroxide (H_2_O_2_) to oxygen and water; therefore, the activity of CAT was evaluated according to the exponential decomposition of H_2_O_2_ at 240 nm. Results are expressed in terms of units per milligram of protein. A method by Nandi and Chatterjee was followed to assay superoxide dismutase (SOD) levels in liver homogenates (Nandi and Chatterjee, 1988). This method utilizes the inhibitive ability of SOD on autooxidation of pyrogallol (1,2,3-benzentriol) at an alkaline pH. A method by Hillegass et al. (1990) was used to determine myeloperoxidase (MPO) activity by measuring peroxidase activity of MPO that catalyzes the oxidation of peroxide. The amount of MPO required to degrade 1 µM of peroxide/min describes one unit of MPO. Peterson modified-Lowry’s method was used to evaluate total protein content in liver homogenates (Peterson, 1977). UV-160-Shimadzu recording spectrophotometer was used to record absorbances. Malondialdehyde (MDA) level was assayed spectrophotometrically by measuring the product of MDA reaction with thiobarbituric acid (TBA), a pink complex, at 535 nm (Uchiyama and Mihara, 1978). To determine liver homogenate content of protein carbonyl (P. carbonyl), a method by Reznick and Packer (1994) was followed. This method is based on the reaction of the carbonyl group with 2,4-dinitrophenylhydrazine (DNPH) to form a spectrophotometrically detectable hydrazone product at 370 nm. The results are expressed as nanomoles of carbonyl group per milligram of protein, with a molar extinction coefficient of 22,000 M/cm.

### TUNEL Assay

TUNEL assay was performed for the purpose of assessing apoptosis. Liver sections (4 µm) were deparaffinized and subjected to subsequent gradual hydration prior to staining. ApopTag peroxidase *In Situ* Apoptosis Detection kit was used according to the manufacturer’s instructions (Serological Corporation, Norcross, USA). DNA fragmentation, a key indicator of apoptosis, is detected using this kit. Cell death was confirmed using M30 CytoDeath monoclonal antibodies by detecting the caspase-cleaved fragment of cytokeratin18.

### Immunohistochemical Staining

Mounted sections were immersed in sodium citrate buffer (0.1 M, pH 6) and placed in a water bath for 15 min to unmask antigen epitopes. Afterwards, sections were incubated with 0.3% H_2_O_2_ in methanol to block nonspecific binding to endogenous peroxidase. Sections were incubated overnight at 4°C with rabbit anti-rat primary antibodies, anti-COX-2, anti-iNOS, anti-NF-_k_B-P65, and anti-Ki-67; in addition to M30 CytoDeath, monoclonal ED-2 anti-rat antibody, and polyclonal anti-rabbit antibodies, anti- GST-p and anti-p-TNFR. After incubation, slides were washed with PBS and incubated with polyvalent biotinylated goat-anti-rabbit, a secondary antibody, for 10 min at room temperature (1:200 dilution). Universal LSAB kit and DAB plus substrate kit were both used to perform a standard staining protocol. Hematoxylin was used in additional counter-staining. Slides were observed under an Olympus DP71 optical microscope, and tissue images were obtained.

### Histone Deacetylase Activity Assay

HDAC Colorimetric Assay Kit (Millipore Corporation, 28,820 Single Oak Drive, Temecula, CA 92590, Catalog number: 17-374) was used to measure HDAC activity in liver homogenate.

### Determination of Tumor Necrosis Factor-α

TNF-α level in serum was quantitatively measured using ELISA (Chessum et al., 2015), according to the manufacturer instructions (ELISA kits; R&D Systems, Minnesota, USA). Results are presented in picograms per milligram.

### Cell Culture

Human liver carcinoma cell lines (HepG2) were obtained frozen in liquid nitrogen from the American Type Culture Collection (ATCC). HepG2 were grown as “monolayer culture” in RPMI 1640 medium (HyClone, USA) and 1% of 100 U/ml penicillin and 100 μg/ml streptomycin (Sigma, USA) supplemented with 10% fetal bovine serum (Sigma) at 37°C in a humidified 5% CO_2_ atmosphere. Cells were sub-cultured each 4–6 days using trypsin 0.25% (HyClone).

### Cell Viability Assay

HepG2 cells were seeded in 96-well plates at the density of 10,000 cells/well and grown in 100 µl of complete growth medium. Complete growth medium was replaced by serum-free medium after cells were allowed to attach for 24 h, after which cells were incubated for at least 12 h. Cells were incubated for 24 h after treatment with various concentrations of safranal (1, 0.3, 0.1, 0.03, 0.01 mM) prepared from 10 mM stock solution. After the incubation period, the viability of HepG2 cells was assessed using Cell Titer-Glo luminescent cell viability assay kit according to manufacturer’s instruction (Promega, 2800 Woods Hollow Rd., Madison, Wisconsin, USA).

### Caspase-3 and -7 Assay

HepG2 cells were seeded in 96-well plates at the density of 10,000 cells/well and grown in 100 µl of complete growth medium. Complete growth medium was replaced by serum-free medium after cells were allowed to attach for 24 h, after which cells were incubated for at least 12 h. Cells were incubated for 48 h after treatment with various concentrations of safranal (1, 0.7, and 0.5 mM) prepared from 20 mM stock solution. After the incubation period, caspase-3 and -7 activities were measured using Caspase-Glo 3/7 luminescent assay kit according to manufacturer’s instruction (Promega G8091). Luminescent signal was detected using GloMax Discover System (Promega). The induction of DNA double-strand breaks (DSBs) was assessed by measuring phosphorylation of the histone H2X using western blotting.

### ELISA

Supernatant of safranal-treated cells were used to investigate the effect of safranal on IL-8 (CXCL8) secretion level. Human IL-8 ELISA Kit (EZHIL8, Millipore, USA) was used according to manufacturer’s instructions. Absorbance was recorded at 450 nm with background subtraction at 570 nm using a microplate reader (Biotek, Winooski, VT, USA).

### Statistical Analysis

SPSS (version 20) statistical software (SPSS Inc., Chicago, IL, USA) and GraphPad Prism 5 (GraphPad Software, San Diego, CA, USA) were used for statistical analysis and plotting graphs. Values are expressed as mean ± SEM of six rats per group. A one-way study of variance (Benassi et al., 2021), followed by post hoc Dunnett’s test, was used to compare the differences among the groups. All *p*-values less than 0.05 (*p* < 0.05) were considered as statistically significant.

## Results

### Safranal Inhibits DEN/2-AAF–Induced FAH Formation and GST-p Expression

**FIGURE 2:**
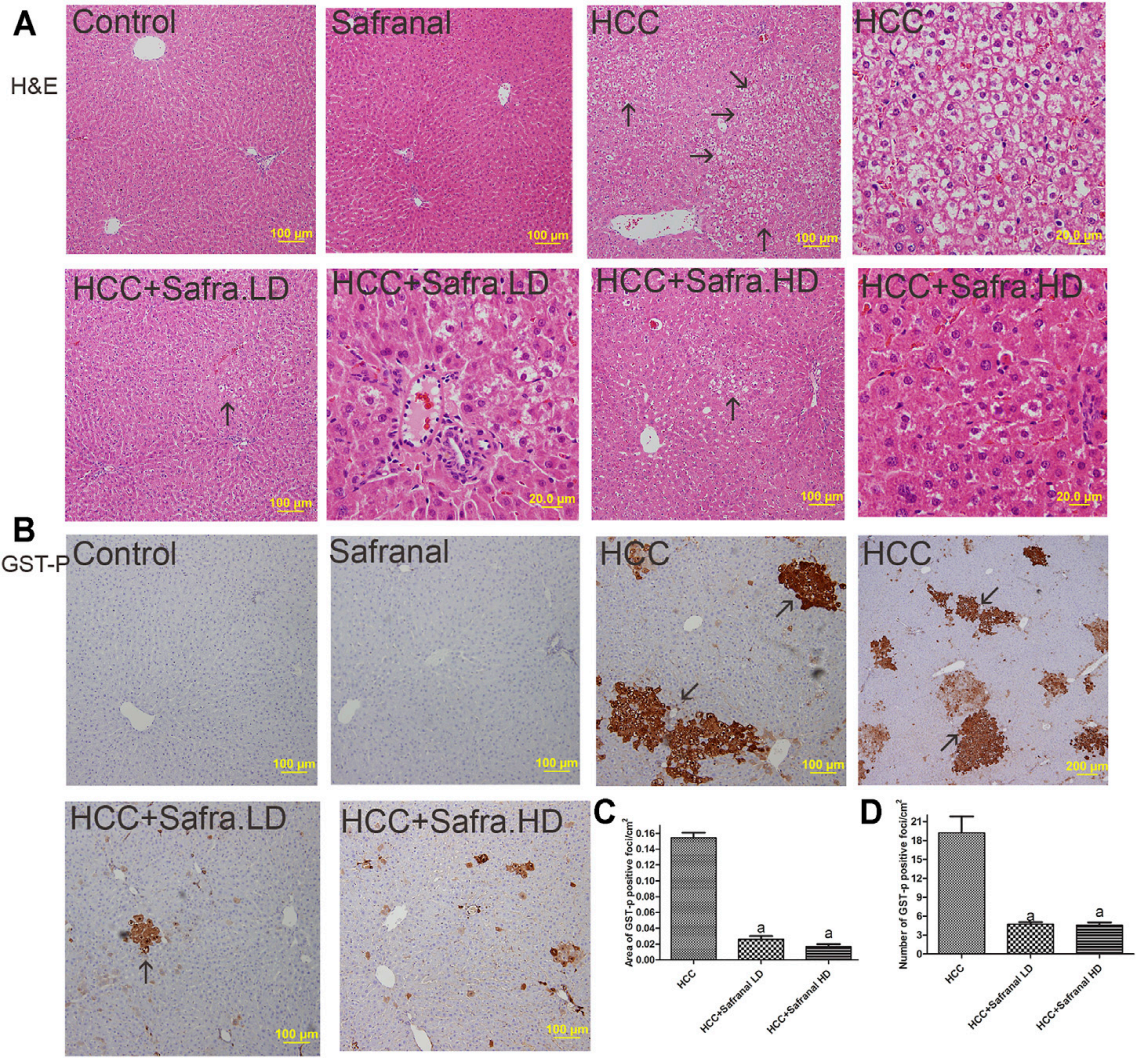
Safranal inhibits DEN/2-AAF–induced induction of AHF **(A)** and of GST-p expression **(B–D)**. **(A)** Figures representing the livers of all groups with a magnification of ×100 and ×400 (scale bars = 100 and 20 μm) using H&E staining. AHF is indicated by arrows at ×100 magnification (scale bar = 100 μm). **(B)** Representative Images of immunohistochemical stains with GST-p of in all groups studied. The brown color represented those cells and is indicated by arrows. **(C, D)** Present quantitative analyses in 10 fields of each section of the GST-p–positive foci and quantitative region analysis of the GST-p–positive foci ×100 magnifications. Treatment of HCC rats with safranal decreased the number and area (mm^2^) of GST-positive foci. The value was evaluated by one-way ANOVA followed by Dunnett’s *t*-test: ^a^ *p* < 0.05 vs. HCC group. Data are represented as mean ± SEM of six animals per group.

Figure 2 shows the classical FAH in the livers of the cancer group (Figure 2A), which are represented by the presence of large, irregular, and pale hepatocytes as well as extensive cytoplasmic vacuums with large hyperchromatic nuclei. The number and size of these foci were remarkably decreased in the protective groups treated with low/high doses of safranal (Figure 2A).

An increase in the number and area of placental glutathione S-transferase (GST-p) in the liver is a reliable marker of HCC induced with carcinogens in the liver. Figure 2B shows that, compared with the control group, the GST-p protein expression is significantly increased as reflected by the area per square centimeter and the number of foci in the livers of the HCC group. The number of GST-p positive foci and area per square centimeter increased significantly in animals treated with DEN/2-AAF. However, safranal treatment alone did not induce formation of such foci. GST-p was reduced in the livers of rats treated with safranal before cancer development compared with rats that were given only carcinogens.

### Safranal Induces Apoptosis, Inhibits Proliferation, and Decreases HDAC Activity in Rats With HCC

**FIGURE 3:**
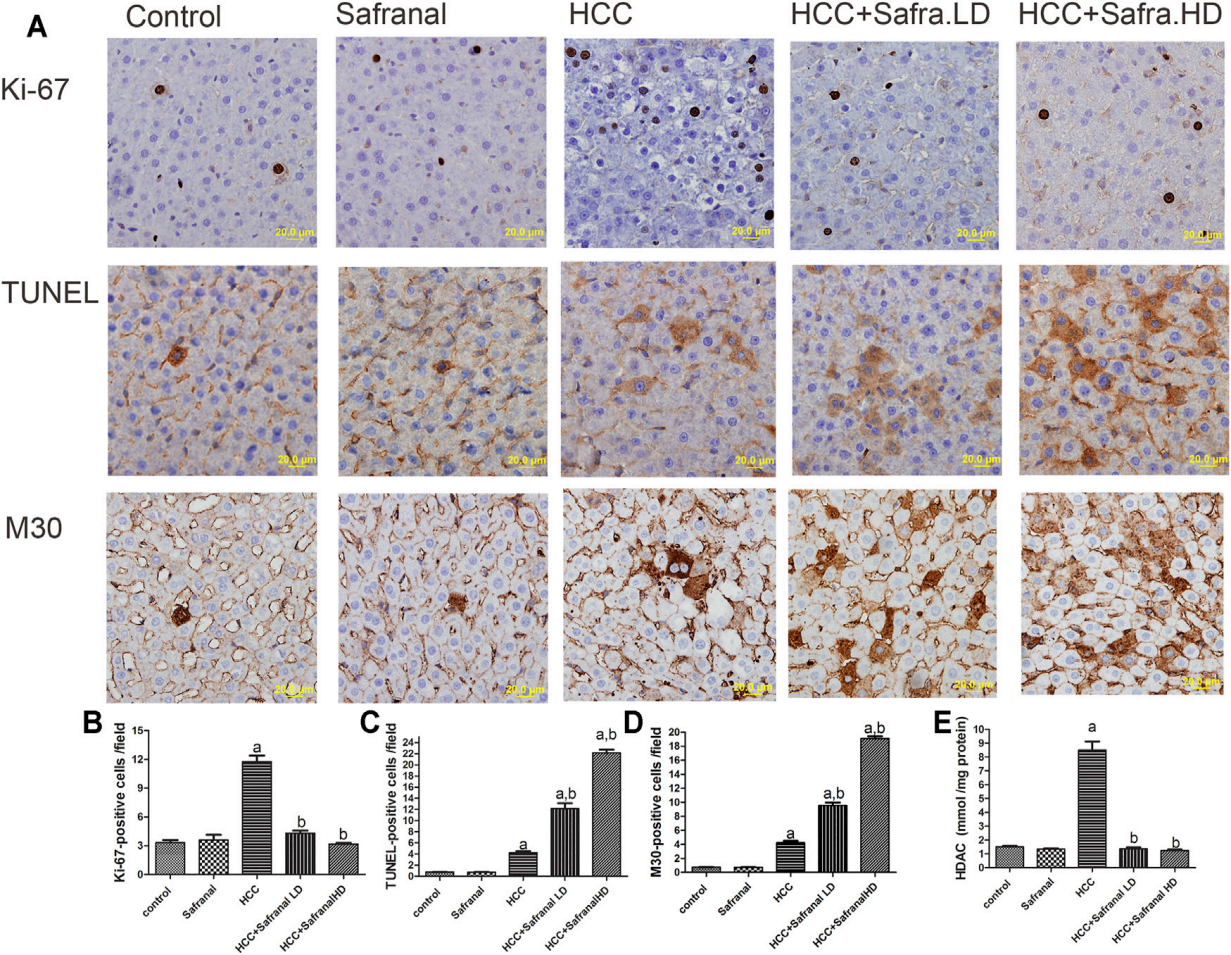
Effects of safranal on proliferation (Ki-67) and apoptotic cell death (TUNEL and with M30) and HDAC activity in rat livers. **(A)** The upper panel includes representative images of immunohistochemical staining with Ki-67, TUNEL, and M30 in liver sections from all the groups (scale bar = 20 µm). The bottom panel represents quantitative analysis of Ki-67 **(B)**, TUNEL **(C)**, and M30 **(D)** positive cells as well as HDAC activity **(E)**, which is expressed in mmol/mg protein. The positive expression in each section was calculated by counting the number of brown staining in 10 fields at ×400 magnifications, then the number of positive cells/field. Values expressed as mean ± SEM for six animals in each group. Significance was determined by one-way ANOVA followed by Dunnett’s *t*-test: ^a^ *p* < 0.05 vs. control group, ^b^ *p* < 0.05 vs. HCC group.

The nuclear Ki-67 is a marker of cell division and its overexpression is an indicator of tumorigenesis (Scholzen and Gerdes, 2000). DEN-2AAF treatment caused a significant increase in the number of Ki-6–expressing cells in the livers of HCC rats compared with those of the control group (Figure 3A,B). Treatment with safranal alone did not induce a significant change in the number of Ki-67–expressing hepatocytes. However, safranal treatment reduced the protein expression of this protein in the livers of rats treated with DEN-2AAF after the administration of safranal.

Given the importance of programmed cell death (apoptosis) in the prevention and treatment of liver cancer, apoptosis was assessed here by estimating numbers of TUNEL-positive cells and M30 CytoDeath-positive cells in animal livers. Interestingly, cell death represented by the number of TUNEL and M30 CytoDeath-positive cells increased significantly in the livers of HCC rats compared with the control group. The percentage of apoptotic cells increased in the HCC rats that were pre-treated with safranal. Such increase in TUNEL-positive cells and M30 CytoDeath-positive cells was significant compared with that of HCC group and the control group. However, there was no increase in these markers in normal rats treated with a high dose of safranal in comparison with the control group.

It is equally interesting that the livers of rats treated with DEN-2AAF had a significant increase in the activity of histone deacetylase (HDAC) enzyme compared with the control group (Figure 3E). Although when administered alone did not affect the HDAC activity in the livers of normal rats, safranal had the ability to reduce HDAC activity in the livers of HCC group in a non–dose-dependent manner (Figure 3E).

### Safranal Reduces Oxidative Stress and Enhances Antioxidant Capacity in Livers of DEN-2-AAF–Treated Rats

As shown in Table 1, levels of MDA, and P. carbonyl and MPO activity increased significantly in the cancer group (HCC) and were accompanied by a decrease in the activity of CAT and SOD enzymes. In contrast to these dramatic changes in oxidative stress markers in the cancer model, there were improvements in those indicators in the livers of rats treated with low/high doses of safranal compared with the results of the HCC and the control groups. Interestingly, these effects were dose dependent, as rats of a high safranal dose alone did not affect any of these results in normal rats and stayed at control treated with high dose showed results that were very close to those of the control group.

**TABLE 1:** Effect of safranal on oxidative stress markers

Groups	MDA	P. carbonyl	CAT	SOD	MPO
Control	0.66 ± 0.02	1.51 ± 0.04	1.51 ± 0.04	4.07 ± 0.04	33.55 ± 0.31
Safranal (Safra)	0.64 ± 0.02	1.52 ± 0.03	1.52 ± 0.03	4.05 ± 0.09	32.61 ± 1.61
HCC	0.87 ± 0.02^a^	2.16 ± 0.06^a^	2.16 ± 0.06^a^	3.38 ± 0.06^a^	49.92 ± 4.5^a^
HCC + Safra LD	0.61 ± 0.02^b^	1.61 ± 0.06^b^	1.61 ± 0.06^b^	3.96 ± 0.13^b^	27.47 ± 1.77^b^
HCC + Safr^a^ HD	0.63 ± 0^.^03^b^	1.45 ± 0.06^b^	1.45 ± 0.06^b^	4.02 ± 0.8^b^	31.54 ± 2.4^b^

Values are expressed as mean ± SEM of six rats per group. Concentration is expressed as nmol/mg protein for MDA, P. carbonyl activity is expressed as unit/mg protein for CAT, and SOD activity is expressed as m unit/mg protein for MPO. Significance was determined by one-way ANOVA followed by Dunnett’s *t* test:

^a^ *p* < 0.05 vs. normal group.

b *p* < 0.05 vs. HCC.

### Safranal Reduces the Upregulation of Liver Tissue Expressions of ED-1, ED-2, and p-TNF-R1 and Increases the Serum TNF-α Concentration in DEN/2-AAF–Treated Rats

**FIGURE 4:**
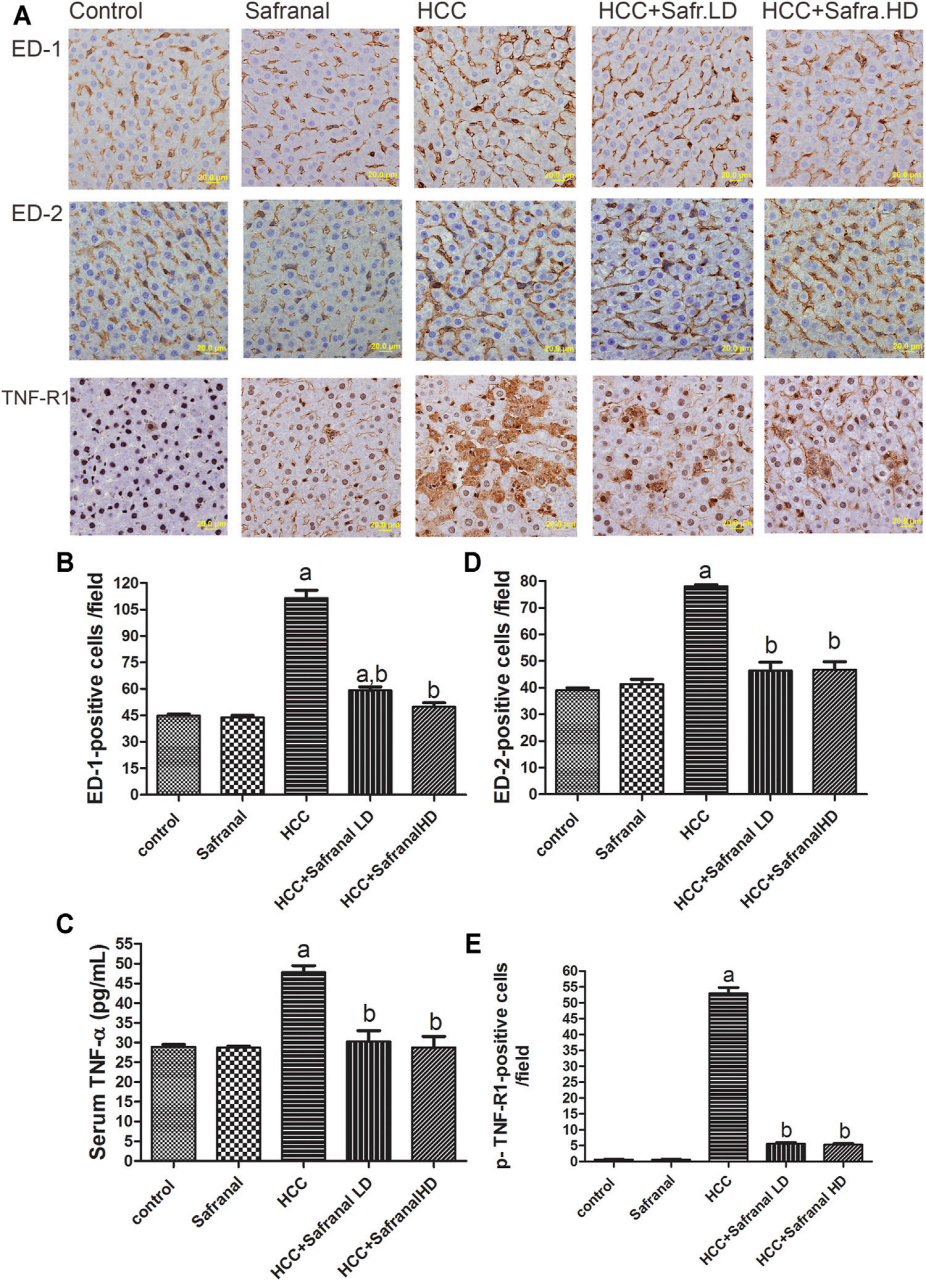
Safranal reduces the upregulation of ED-1, ED-2, and p-TNF-R1 in liver and TNF-α in serum of HCC rats. **(A)** The upper panel are representative images of immunohistochemical staining with ED-1, ED-2, and p-TNF-R1 in the liver section from all the groups. **(B**–**D)** The positive expression of cells in each section was calculated by counting the number of brown staining in 10 fields at ×400 magnifications then the number of positive cells/fields. **(E)** Shows quantitative analyses of TNF-α in serum. Data are represented as mean ± SEM for six animals in each group. Significance was determined by one-way ANOVA followed by Dunnett’s *t*-test: ^a^ *p* < 0.05 vs. control group, ^b^ *p* < 0.05 vs. HCC group.

Expression of both ED-1 and ED2 is considered a cytometric marker to assess the activity of macrophages and resident macrophages (Kupffer cells) (Atretkhany et al., 2016; Taniguchi and Karin, 2018). Figure 4 shows that there was a significant increase in the number of microphages in the livers of rats treated with DEN-2-AAF compared with the control group. This increase in the number of microphages decreased significantly in the rats treated with safranal at low/high doses (Figure 4A–C). Once again, safranal alone had no effect on the expression of ED1 and ED2 compared with control. There was a significant increase in serum TNF-α concentration and number of p-TNF-R1–positive cells in the livers of HCC group compared with the results of the control group (Figure 4D,E). Safranal alone had no significant impact on TNF-α and p-TNF-R1 expressions in normal rats. However, when administered to rats with cancer, safranal reduced both TNF-α level in serum and p-TNF-R1 expression in liver.

### Safranal Reduces the Upregulated Expressions of NF-κB p65, COX-2, and iNOS in Livers of DEN/2-AAF–Treated Rats

**FIGURE 5:**
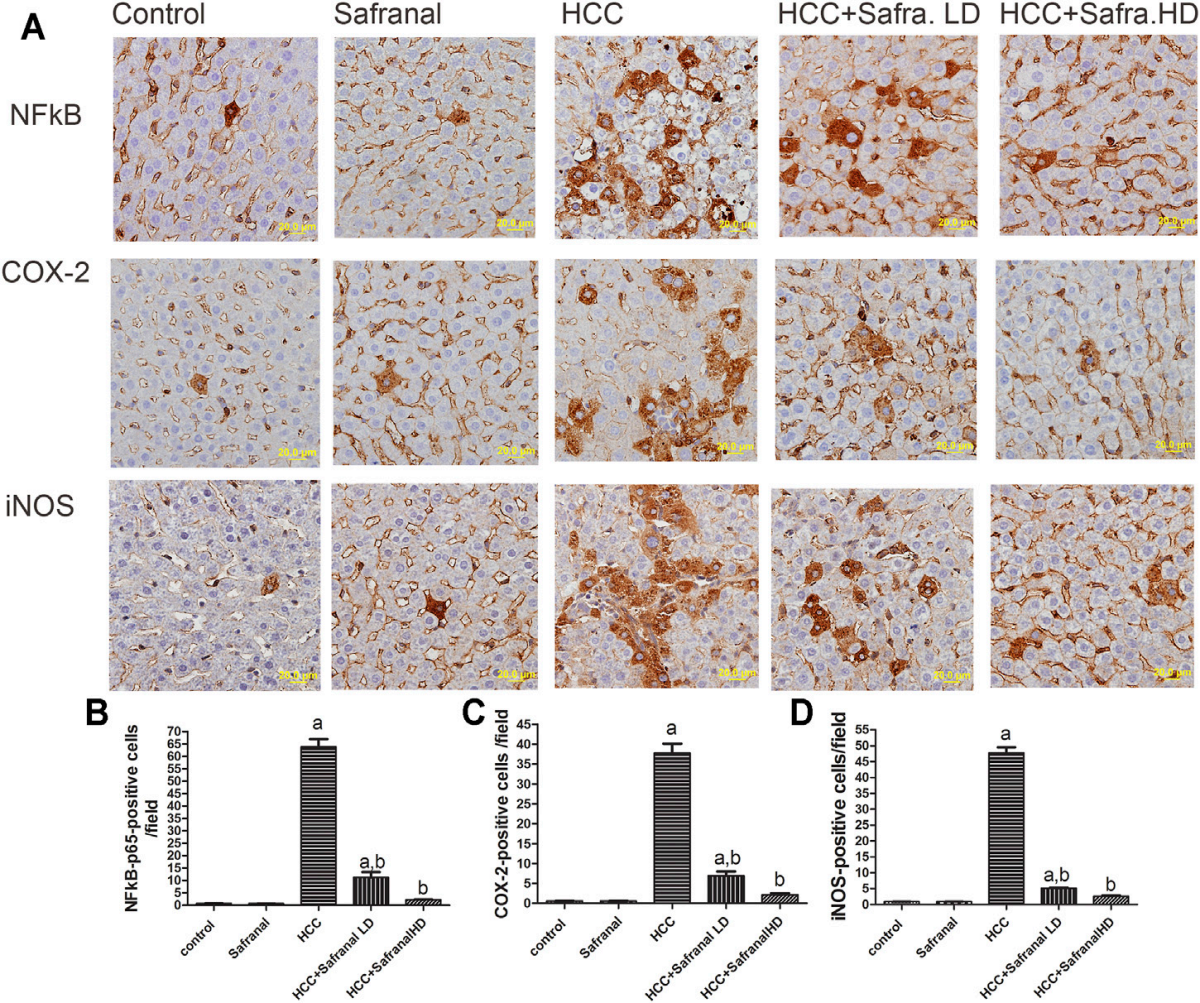
Safranal inhibits DEN/2-AAF–induced upregulation of NF-kB-p65 and COX-2 and iNOS-positive cells expressions. **(A)** The upper panel are representative images of immunohistochemical staining with NF-kB-p65, COX-2, and iNOS in the liver section from all the groups (scale bar = 20 µm). **(B, C, D)** show quantitative analyses of NF-kB-p65, COX-2, and iNOS-positive cells. The positive expression of cells in each section was calculated by counting the number of brown staining in 10 fields at ×400 magnifications then the number of positive cells/fields. Data are represented as mean ± SEM of six rats per group. Significance was determined by one-way ANOVA followed by Dunnett’s *t*-test: ^a^ *p* < 0.05 vs. control group, ^b^ *p* < 0.05 vs. HCC group.

Figure 5 shows that DEN-2-AAF treatment caused a significant increase in the number of both of NF-kB-p65-, COX-2-, and iNOS-positive cells mostly in hepatocytes around the central vein and in Kupffer cells. Pre-treatment with low/high doses of safranal almost completely abolished the effects of DEN-2-AAF in comparison with HCC group (Figure 5).

### *In Vitro* Analyses

**FIGURE 6:**
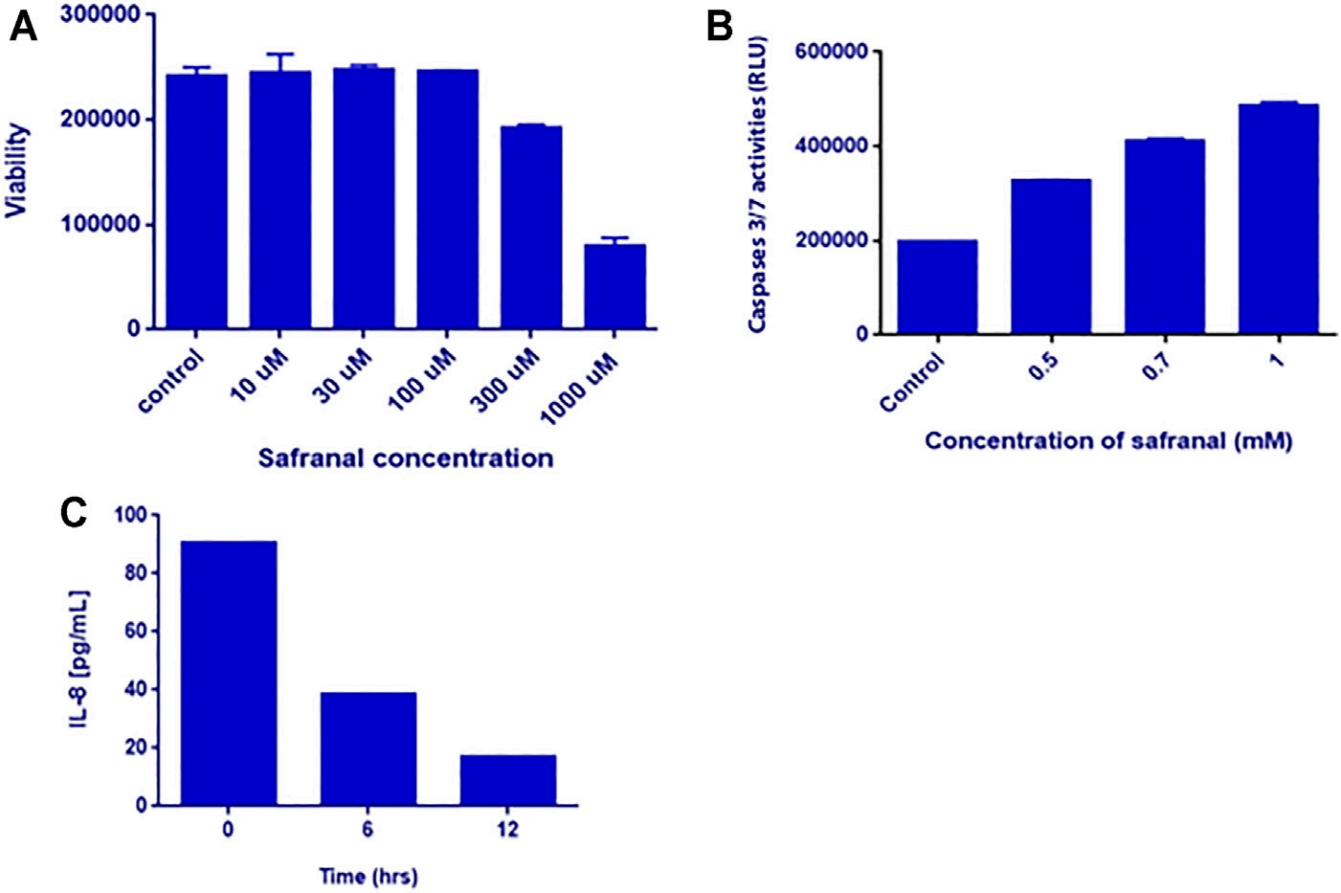
*In vitro* analysis. **(A)** Viability of HepG2 cells after safranal treatment for 24 h. HepG2 cells were treated with 0.01, 0.03, 0.1, 0.3, and 1 mM of safranal. **(B)** Caspase-3/7 activities after safranal treatment for 48 h. HepG2 cells were treated with 0.5, 0.7, and 1 mM of safranal. **(C)** IL-8 secretion after safranal treatment. HepG2 cells were treated with 2 mM for 6 and 12 h, and subsequently, the supernatants were analyzed by IL-8 ELISA.

*In vitro* analysis was performed to highlight the anticancer effects of safranal on HepG2 cells. Various concentrations of safranal (0.01, 0.03, 0.1, 0.3, 1 mM) were used to treat the cells for 24 h. Cell viability was assessed using Cell Titer-Glo kit. Safranal exhibited a significant dose-dependent reduction of HepG2 cell viability. At a concentration of 1 mM, safranal was able to reduce cell viability by almost 70% (Figure 5A). Following the Post treatment with various concentrations of safranal for 48 h, a significant increase in caspase-3 and -7 activities was noted at a concentration of 1 mM (Figure 5B). A dramatic decrease in IL-8 secretion as early as 6 h was also reported when HepG2 cells were treated with various concentrations of safranal (Figure 6C).

## Discussion

In the continuation of our previous studies to evaluate the anticancer effect of safranal both *in vivo* and *in vitro* (Al-hrout et al., 2018; Amin, 2020; Amin, 2021; Amin et al., 2021), here we investigate safranal potential to prevent liver cancer in a drug-induced HCC animal model. The DEN’s two-step HCC animal model represents the onset of tumor cell formation by DEN and its stimulation by 2-AAF causes pathophysiological changes that mimic human liver cancer (Tolba et al., 2015; Santos et al., 2017). This model enabled us to investigate mechanisms that safranal may utilize during the early stage of stimulating liver cancer and their relationship to oxidative stress and inflammation in livers of HCC-bearing rats.

As reported here, using DEN and 2-AAF caused paraneoplastic changes in livers of rats. Those histopathological changes included the development of FAH. Paraneoplastic cells in these FAH are characterized by cell irregularity, multinucleation, lack of cytoplasm, and large size of the nucleus relative to the cytoplasm. Administering safranal to HCC-induced rats decreased the incidence of FAH. HCC-induced rats also showed an increase in the area and number of GST-p positive foci, a reliable and sensitive marker of pre-neoplastic foci and nodules in liver cancer (Tew et al., 2011; Satoh, 2018). Safranal’s inhibition of pre-neoplastic lesions and FAH was accompanied by a significant decrease in both the number and area of GST-p–positive foci in HCC groups. These results confirm that safranal is a promising candidate in cancer chemoprevention by acting against early, pre-neoplastic liver events that promote HCC progression.

One of the important changes induced by DEN and 2-AAF in the liver of rats is the presence of a dramatic increase in cell division represented by the Ki-67 marker (Scholzen and Gerdes, 2000). In this study, the safranal caused inhibition of the number of Ki-67–positive cells in DEN-treated animals, which confirms further inhibition of cell proliferation in the livers of carcinogenic rats under the influence of the safranal treatment. In HCC-induced animals, a significant increase in apoptotic cell death was shown and was represented by an increased number of TUNEL- and M30 CytoDeath-positive cells, both of which are indicators of DNA fragmentation and early apoptosis, respectively. Induced apoptosis could compensate for the increased cell proliferation in HCC-induced groups. Continued development as a result of loss of apoptotic mechanisms in association with the increased cell proliferation are main physiological changes in developing cancer (Maru et al., 2016; Zhou et al., 2016). Inhibiting cell proliferation and inducing apoptotic activity is consistent with what has been reported in alveolar human cancer lung cell line, human prostate cancer cell line, and in human liver cancer (HepG2) and colorectal cancer cells (Samarghandian and Boskabady, 2012; Samarghandian and Shabestari, 2013; Al-hrout et al., 2018). In these experiments, safranal activated DNA double strand breakage damage and activated pro-apoptotic effects through the activation of both intrinsic and extrinsic initiator caspases, indicating endoplasmic reticulum (ER) stress-mediated apoptosis. Similar results were confirmed here in the hepatocyte cell model HepG2, where the ability of safranal to increase apoptosis was mediated by caspase-dependent increased abundancy (caspase-3 and -7 activities) and mediated by activated DNA double strand damage (upregulated p-H2AX protein level as assayed by immunoblotting). Thus, the present results indicate that the safranal-induced inhibition of hepatic neoplasia was mediated by both upregulation of apoptosis and downregulation of cellular proliferation.

A wide range of cellular changes resulting from oxygen stress, such as cellular DNA damage and inflammation, are strongly associated with the occurrence of cancer. In addition, genetic instability and higher cell proliferation contribute to the development of FAH and its progression to adenomas and HCC (Reuter et al., 2010; Marra et al., 2011). In this study, DEN/2-AAF stimulated an increase of several indicators of hepatic oxidative stress such as MDA and P. carbonyl. They also increased the hepatic MPO, which is an indicator of oxidative stress and inflammation (Loria et al., 2008). In addition and in agreement with previous observations in HCC model, the livers of HCC rats in this study showed that the depletion of endogenous antioxidants such as SOD and CAT were responsible for reversing the ROS-induced oxidative damage (Marra et al., 2011; Hamza et al., 2018; Hamza et al., 2021). On the contrary and in HCC-induced animals, the administered saffron attenuated the changes of antioxidants in the livers of rats given nitrosamines, and this improvement was accompanied by reduction of oxidized lipid (MDA) and protein (P. carbonyl). It also upregulated H2AX protein level, a sensor for DNA double strand breaks *in vitro* hepatocyte cell model (HpG2), as markers of oxidative stress in DNA (Al-hrout et al., 2018). Recent studies reported an anti-oxidant effect of safranal as represented by decreasing the oxidative stress, assessed by MDA, and by restoring normal levels of anti-oxidant enzymes including SOD and CAT (Hamza et al., 2015; Cerdá-Bernad et al., 2020).

Chronic inflammation plays an important role in the onset and progression of cancer (Taniguchi and Karin, 2018). This cross-talk between chronic inflammation and cancer includes activation of immune cells such as macrophages, Kupffer cells, and neutrophils, and the production of pre-inflammatory mediators including COX-2 and iNOS, and of transcription factors, like the nuclear transcription factor kappa B (NF-κB) (Reuter et al., 2010; Atretkhany et al., 2016; Taniguchi and Karin, 2018). In many human cancer tissues and in cancer induced by chemicals such as DEN, the development of cancer has been associated with an increase in macrophage cells and neutrophils, as well as an increase in the expression of TNF-α and its receptor TNFR-1 (Roberts et al., 2007; Hayden and Ghosh, 2014; Amin et al., 2016; Hamza et al., 2021). TNF-α is mainly produced by activated immune cells such as macrophages and neutrophils, and then triggers other pro-inflammatory cytokines (Balkwill, 2009; Atretkhany et al., 2016). TNFR1 activation by TNF-α has tumor-promoting action, which is associated with proliferation and activation of NF-κB (Roberts et al., 2007). In the present work, treating HCC-induced rats with safranal alleviated cancer-associated inflammation by reducing the number of both hepatic ED1- and ED2-stained microphages and restoring normal hepatic MPO levels, a marker of neutrophil infiltration (Loria et al., 2008), as well as inhibiting the TNF-mediated inflammatory pathway via reducing the content of TNF-α and the number of p-TNF-R1–positive cells.

IL-8 is a chemokine produced by macrophages that induces a series of physiological responses required for migration and phagocytosis. In HepG2 cells, safranal reduced the concentration of IL8 in a time-dependent manner. This decrease in inflammatory cell infiltration and TNF-α was associated with the inhibition of the protein expression of COX-2, a pro-inflammatory enzyme involved in PG production, and iNOS, the key enzyme in NO production. This indicates that the protective effects of safranal against carcinogenesis could be mediated by decreasing inflammation through downregulation of COX-2 and iNOS. In this regard, the anti-inflammatory effect of safranal was studied in a recent study in which saffron dose-dependently decreased iNOS and COX-2 levels in lipopolysaccharide-stimulated RAW264.7 cells and bone marrow–derived macrophages (Lertnimitphun et al., 2019). Safranal also reduced the production of IL-6 and TNF-α in RAW264.7 cells, and this was accompanied by a reduction in the phosphorylation and expression of NF-ĸB signaling pathway proteins (Lertnimitphun et al., 2019).

NF-kB is considered as one of the most powerful players involved in the development of many types of cancer, and it is characterized by linking chronic inflammation and oxidative stress to the tumorigenesis and inflammation associated with cancer (Hayden and Ghosh, 2014; Taniguchi and Karin, 2018). Inflammatory-associated cancer has been found to be active by increasing oxidative stress and upregulation of inflammatory markers including NF-κB and iNOS (Xia et al., 2014). NF-κB plays a major role in promoting cancer by modulating the expression of many genes via nuclear oxidative stimuli. This modulation in gene expression by NF-kB is responsible for altered inflammatory responses, upregulation of COX-2 and iNOS, promoting cell proliferation, and inhibiting cell death (Hayden and Ghosh, 2014; Taniguchi and Karin, 2018). In the present work, safranal treatment decreased oxidative stress, which was accompanied with inhibition of inflammatory markers including COX-2, iNOS, and NF-κB. The decrease of Kupffer cells and neutrophils reported here seems to be associated with an early inactivation of the NF-kB signaling pathway, as reflected in the early *in vitro* inhibition of p-IkB and IL-8. The findings reported here suggest that safranal’s anti-cancer properties could be attributed to its anti-inflammatory activities through downregulation of NF-κB, COX-2, and iNOS expression levels as well as to the reduction of both TNF-α and its receptor TNFR1.

The process of regulating gene expression by epigenetic modulators has been introduced as a novel mean of targeted therapy in the fight against cancer (Chessum et al., 2015). One of the target enzymes is histone deacetylase (HDAC), which is responsible for packing DNA tightly around histones by removing acetyl groups from an ε-N-acetyl lysine amino acid on histones, minimizing the chance of RNA polymerases contacting DNA and resulting in decreased gene expression (Park and Kim, 2020). HDACs play a major role in the onset and development of cancer by removing acetyl groups from histones that are involved in the regulation of the cell cycle, apoptosis, the DNA-damage response, metastasis, angiogenesis, and autophagy (Li and Seto, 2016). Results presented here showed a significant effect of safranal pre-treatment on inhibiting the increased HDAC expression in a DEN-treated HCC model and restoring it to control levels. Taken together, these findings suggest that anti-proliferative and pro-apoptotic properties of safranal could be attributed, at least in part, to its inhibitory ability of HDAC overexpression in cancer.

## Conclusion

Findings reported in this study showed the potent efficacy of safranal against drug-induced HCC *in vivo*. Safranal treatment was efficient in inhibiting FAH formation in DEN-induced HCC models, restoring the antioxidant normal levels, and reducing all tested oxidative stress markers. In addition, significant decreases in the activity of inflammatory markers, COX-2, iNOS, NF-_k_B, TNF-α, and its receptor p-TNF-R1 were observed in DEN-induced HCC model pre-treated with safranal. Moreover, pre-treatment with safranal induced a reduction in the number of Kupffer cells and macrophages. These findings were also confirmed *in vitro* by utilizing the human hepatoma cell line “HepG2” where safranal has consistently demonstrated pro-apoptotic and anti-inflammatory properties.

## Data Availability

The raw data supporting the conclusions of this article will be made available by the authors, without undue reservation.
